# Soapwort (*Saponaria officinalis* L.) Extract vs. Synthetic Surfactants—Effect on Skin-Mimetic Models

**DOI:** 10.3390/molecules26185628

**Published:** 2021-09-16

**Authors:** Ilona Jurek, Aleksandra Szuplewska, Michał Chudy, Kamil Wojciechowski

**Affiliations:** 1 Faculty of Chemistry, Warsaw University of Technology, Noakowskiego 3, 00-664 Warsaw, Poland; ijurek@ch.pw.edu.pl (I.J.); aszuplewska@ch.pw.edu.pl (A.S.); chudziak@ch.pw.edu.pl (M.C.); 2 SaponLabs Ltd., Noakowskiego 3, 00-664 Warsaw, Poland

**Keywords:** saponins, liposomes, keratinocytes, HaCaT, melanoma cells, A375, albumin test, zein test

## Abstract

Our skin is continuously exposed to different amphiphilic substances capable of interaction with its lipids and proteins. We describe the effect of a saponin-rich soapwort extract and of four commonly employed synthetic surfactants: sodium lauryl sulfate (SLS), sodium laureth sulfate (SLES), ammonium lauryl sulfate (ALS), cocamidopropyl betaine (CAPB) on different human skin models. Two human skin cell lines were employed: normal keratinocytes (HaCaT) and human melanoma cells (A375). The liposomes consisting of a dipalmitoylphosphatidylcholine/cholesterol mixture in a molar ratio of 7:3, mimicking the cell membrane of keratinocytes and melanoma cells were employed as the second model. Using dynamic light scattering (DLS), the particle size distribution of liposomes was analyzed before and after contact with the tested (bio)surfactants. The results, supplemented by the protein solubilization tests (albumin denaturation test, zein test) and oil emulsification capacity (using olive oil and engine oil), showed that the soapwort extract affects the skin models to a clearly different extent than any of the tested synthetic surfactants. Its protein and lipid solubilizing potential are much smaller than for the three anionic surfactants (SLS, ALS, SLES). In terms of protein solubilization potential, the soapwort extract is comparable to CAPB, which, however, is much harsher to lipids.

## 1. Introduction

Skin is not only the largest organ of the human body, but it also plays a key role in its functioning. It provides a multifunctional barrier, at the same time separating and linking the human body and the external environment, allowing the body to maintain its internal homeostasis [1]. The human skin is made of epithelial tissue forming the epidermis, and connective tissue forming the dermis and the subcutaneous tissue. The epidermis thickness in various areas of the skin ranges from approximately 0.1 mm around the trunk to roughly 1 mm on the soles of the feet and the hands [2]. The epidermis consists of epithelial cells, called keratinocytes along with Langerhans cells, Merkel cells, and melanocytes, of which the latter three are found in much smaller amounts [3,4]. The epithelial cells undergo continuous keratinization—upon maturation, keratinocytes slowly migrate to the outermost layer of the epidermis (*stratum corneum*), wherein they die and get exfoliated [3,5]. The deeper skin layers (dermis) are populated mostly by fibroblasts, producing and secreting substrates for the connective tissue [6,7]. Their other functions include regulation of epithelial differentiation, formation and deposition of the extracellular matrix. They also promote wound healing and fight against inflammations [3,8].

Proper functioning of the skin barrier relies on a number of complex and interdependent metabolic processes, for example, exfoliation and reconstruction of the epidermis [9]. Any disorder in the functioning of the epidermal barrier may lead to inflammation, with contact eczema being just one example [10]. Many liposoluble substances are capable of penetrating the *stratum corneum*. They can pass the layered structure formed by amphiphilic ceramides and diffuse towards the deeper layers of the skin. On the other hand, mechanical injuries damaging the *stratum corneum*, or a too intense moisturization of the skin, may disturb cohesion between the cells (“bricks”) and the intercellular lipids’ “mortar”, thereby additionally increasing the permeability of the skin’s protective barrier [11,12]. The latter might also be weakened by excessive dryness causing the loss of keratin elasticity and cracking of the epidermis [2,4]. A common cause of increased skin susceptibility to infections and allergies is the use of aggressive cleansing cosmetics, soaps, and other detergents [13,14]. Surface-active molecules used to remove dirt, sweat, sebum and oils from the skin surface also support the process of epidermis exfoliation. Hence, the same properties of surfactants that render them useful for cleansing, may also disturb the proper functioning of the epidermal protective barrier and of the deeper skin layers hosting the living cells [13,15]. All surfactants do interact with lipids and proteins (e.g., keratin) to some extent and are potentially capable of dissolving the lipid membrane components and damaging the skin structure [15]. Commonly used body-washing cosmetics, such as shower gels, liquid soaps, shampoos or bath lotions usually rely on anionic surfactants [16]: SLES (ethoxylated sodium lauryl sulfate), ALS (ammonium lauryl sulfate) or SLS (sodium lauryl sulfate), and their homologs. With increasing awareness of the consumers, the use of the harshest anionic surfactants, such as SLS, clearly ceases, leaving space for milder (yet still synthetic) surfactants. Some representatives of this group include derivatives of amino acids (e.g., sodium lauryl sarcosinate, sodium lauryl glycinate), sugar-based non-ionic surfactants (e.g., lauryl polyglucoside) or amphoteric betaines (e.g., cocamidopropyl betaine or “cocobetaine”).

The synthetic surfactants described above can be relatively easily produced on large or very large scales at reasonable prices. Moreover, they can be conveniently formulated into many products offering the desired stability, texture, and appearance [10]. On the other hand, their production leaves usually high carbon footprints and their chemical harshness and presence of side-product impurities render them often unwanted components of many cosmetic formulations. In this context, natural surfactants produced by living organisms offer an interesting alternative—a combination of high environmental sustainability with unique surface properties, including often exceptionally high surface compression (dilational) viscoelasticity of the adsorbed layers [17]. While mass production of microbial surfactants requires strict biotechnological control and still costly purification (in some processes pathogenic microorganisms are employed), some biosurfactants can also be extracted from macroscopic plants. Although in this case, the production cycle lasts at least several months (or years, e.g., in the case of *Quillaja* trees [18]), the sustainability and carbon footprint are comparable to that of food production.

One of the plants widely distributed around the globe and abundant in biosurfactants is *Saponaria officinalis* L., commonly known as soapwort, a perennial herb f–rom the *Caryophyllaceae* family [19]. The plant produces high amounts of glycosidic compounds (saponins), capable of lowering surface tension to the extent comparable to that of synthetic surfactants [17,20,21]. For centuries, the aqueous extracts of soapwort roots have not only been used as a natural detergent but also as an emulsifier, and even as a medicine to relieve coughing [22,23]. The saponins extracted from soapwort possess also acaricidal [24] and anti-cancer activities [22]. Because of the high affinity to membrane lipids, especially to cholesterol [25,26], some saponins display various levels of hemolytic activity [27]. However, we demonstrated that numerous isolated saponins and saponin-rich extracts do not have any degrading effect on lipid monolayers, as opposed to several synthetic surfactants [21,28,29,30,31,32].

To assure consumer safety of any cosmetic formulation, the effect of its components, especially those displaying pronounced surface activity on the skin, should be characterized to the highest possible degree. This is usually assessed during in vivo tests on human volunteers when the tested substance is applied directly to the skin and the parameters such as hydration, lubrication and smoothness of the skin, or transepidermal water loss (TEWL) are monitored [33]. The human tests should be, however, preceded by simpler assays with simplified models. The EU ban on animal testing forced the development of new alternative methods for assessing the skin-irritation potential [34]. Thus, for preliminary studies of the irritation potential of (bio)surfactants intended for use in cosmetic products, their effect on the model lipid bilayers of liposomes, or cytotoxicity tests towards the cultured skin cells are employed [35,36]. This study aims to compare the irritation potential of four popular synthetic surfactants and the soapwort aqueous extract on human skin models. To this aim, two living cell cultures were employed: normal (HaCaT) and human melanoma cells (A375). The observed differences in cytotoxicity between the synthetic and natural surfactants are interpreted with help of additional experiments for protein (employing the zein solubilization test and binding to bovine albumin) and lipid solubilization. The latter was assessed by comparing the lipid emulsification capacity and liposome solubilization. The test liposomes were composed of a mixture of phospholipid (DPPC) and cholesterol (7:3 mol/mol) and were intended to mimic the cell membrane of human skin cells.

## 2. Results and Discussion

**Figure 1:**
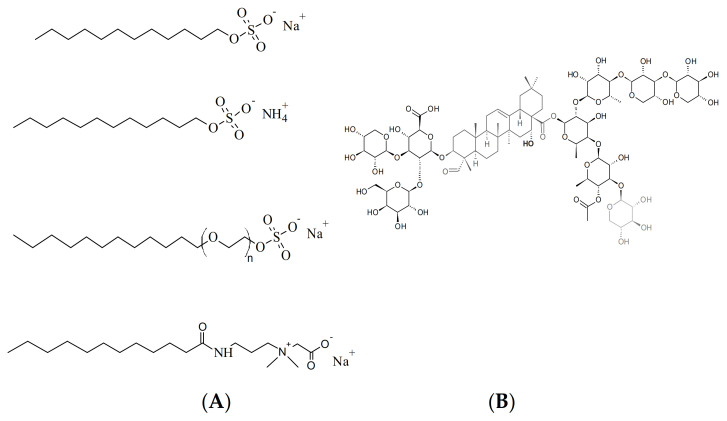
Chemical structures of representative compounds present in: (**A**, from top to bottom) sodium lauryl sulfate (SLS); ammonium lauryl sulfate (ALS); sodium laureth sulfate (SLES); cocamidopropyl betaine (CAPB), and (**B**) soapwort extract (triterpenoid saponins).

The main aim of the present study was to compare the effect of the soapwort extract (SAP) and of four synthetic surfactants (sodium lauryl sulfate (SLS), sodium laureth sulfate (SLES), ammonium lauryl sulfate (ALS) and cocamidopropyl betaine (CAPB)) on viability of model epidermis cells. The general chemical structures of representative components of the tested (bio)surfactants are shown in Figure 1. Two cell lines were used for this purpose: normal human keratinocytes HaCaT and the skin malignant melanoma cells (A375). This allowed us to pinpoint any potential differences in selectivity of the tested (bio)surfactants and their ability to differentiate between the normal and tumor skin cells.

**Figure 2:**
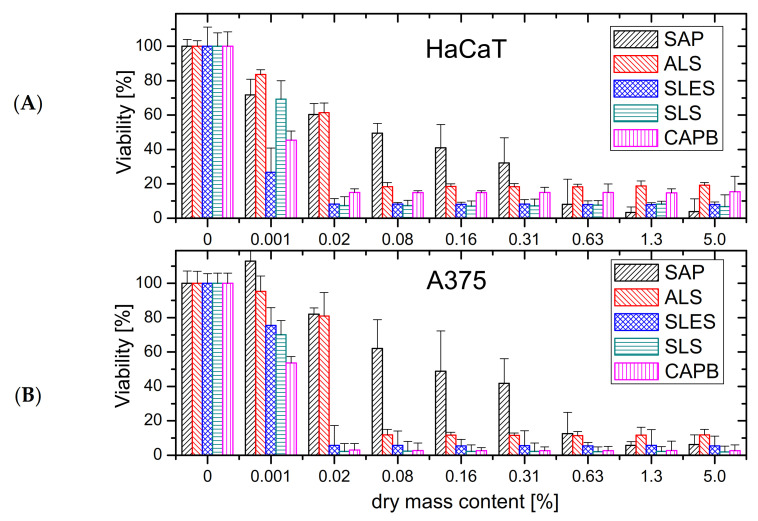
(**A**) The HaCaT (normal keratinocytes) and (**B**) A375 (melanoma cells) viability after 24 h of exposure to sodium lauryl sulfate (SLS), sodium laureth sulfate (SLES), ammonium lauryl sulfate (ALS), cocamidopropyl betaine (CAPB) and a saponin-rich extract from roots of soapwort (SAP).

The cell viability results collected in Figure 2A and Table 1 (half-maximal effective concentration, EC_50_) for the normal HaCaT line clearly differentiate the soapwort extract (SAP) from all the synthetic surfactants. Among the latter group, the anionic SLES and amphoteric CAPB display the strongest cytotoxic effect, both fulfilling the cytotoxicity criterion (viability < 60%) at the dry mass content as low as 0.01 mg/mL (0.001%). Starting from 0.02% of dry mass, cytotoxicity of both the SLES and of its non-ethoxylated analog, SLS, reaches its maximum (viability < 10%). On the other hand, ALS, formally being a simple SLS analog with ammonium counterion instead of sodium, proves almost twice less toxic than SLS above 0.02% and remains the least cytotoxic of the tested synthetic surfactants (>19% viability for all solutions of up to 5% dry mass content). Despite the high initial drop of viability around 0.001%, above 0.02% dry mass the cytotoxicity of CAPB against HaCaT cells remains comparable to that of ALS (viability 15%). For the soapwort extract, the cytotoxicity criterion is fulfilled already above 0.02% dry mass content, but up to 0.31%, the increase in cell toxicity is much less steep. The HaCaT cell viability drops below 20% only above 0.63% dry mass, which is one order of magnitude higher concentration than for ALS. Nevertheless, at the highest concentrations, SAP is even more toxic than SLS or SLES (viability < 5%). The soapwort extract proved less toxic towards the melanoma cells (A375), where the cytotoxicity criterion was not fulfilled below the dry mass content of 0.08 % (Figure 2B, Table 1). Otherwise, the viability profile shows many similarities to that for the normal keratinocytes (Figure 2A). Similar conclusions can be drawn also from the comparison of the results for synthetic surfactants. For example, ALS displays a similarly abrupt increase in cytotoxicity between 0.02% and 0.08% (viability drop from over 80% to merely 15%). At the same time, the latter remains also the least toxic among the synthetic surfactants at higher concentrations.

The less steep viability profiles for SAP are also reflected in significantly higher half-maximal effective concentration (EC_50_) values (Table 1), pointing to a different mechanism of their biological activity. For surface-active (amphiphilic) molecules, cytotoxicity might originate either from simple solubilization of lipids and/or proteins or from biochemical pathways (e.g., specific interactions with receptors). In order to verify whether lipid or protein solubilization might be responsible for the observed differences in cytotoxicity between the four synthetic surfactants and the soapwort extract, below we compared the effect of the tested (bio)surfactants on:

(a) Model liposomes composed of dipalmitoylphosphatidylcholine (DPPC) and cholesterol mixed in a 7:3 mol/mol ratio, which corresponds to a typical phospholipid/cholesterol ratio in mammalian cells, including keratinocytes [37].
(b) Albumin denaturation, by measuring the increase in pH of the bovine serum albumin upon contact with (bio)surfactants.
(c) Zein solubilization, by measuring the percentage of corn zein solubilized by (bio)surfactants.
(d) Oil emulsification capacity using olive oil and engine oil which probes the ability of (bio)surfactants to emulsify lipids.

**Figure 3:**
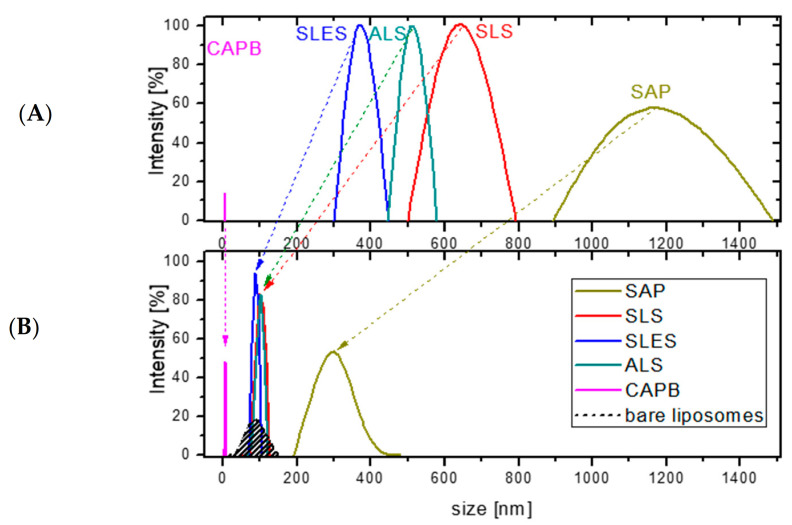
Particle size distribution in 5% (*w*/*w*) solutions of synthetic surfactants SLS, SLES, ALS, CABP and soapwort extract (SAP) in the absence (**A**, top) and presence of DPPC/cholesterol (7:3, mol/mol) liposomes (total lipid concentration 1 × 10^−2^%) (**B**, bottom). The results for bare DPPC/cholesterol (7:3, mol/mol) liposomes are also shown for comparison. All measurements performed in phosphate buffer (pH 7) at room temperature, 2 h after preparation.

**Figure 4:**
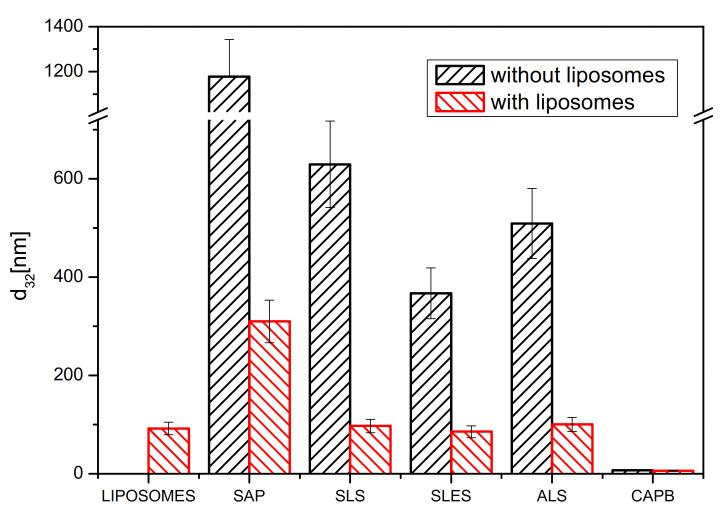
Sauter mean diameter values (d_32_) of the soapwort extract or synthetic surfactants (SLS, SLES, ALS, CPB), 5% concentration of the dry weight in the absence and presence of DPPC/cholesterol (7:3, mol/mol) liposomes, 2 h after preparation. The results for bare liposomes in buffer (in the absence of any (bio)surfactant) are also shown for comparison. All measurements performed at room temperature.

The effect of four synthetic surfactants (SLS, SLES, ALS, CAPB) and the soapwort extract (SAP) on DPPC/cholesterol (7:3 mol/mol) liposomes was investigated in the (bio)surfactant concentration range of 1 × 10^−3^–5% dry weight (the same as employed in the cytotoxicity tests). The reference measurements were performed with the same (bio)surfactants solutions in the absence of liposomes. However, because of the low light scattering intensity for most of the bare (bio)surfactant solutions (references) below 5% dry mass content, in the following, we only discuss the results for the highest dry mass content (see Figure 3 for the size distribution and Figure 4 for the Sauter diameter values, d_32_). The liposome content was set to 0.1 mg/mL and the size distribution in such suspensions was reproducible and stable during at least 120 min, the time necessary to reach equilibrium in the subsequent measurements in mixtures of liposomes and (bio)surfactants. The bare liposome suspensions show a rather narrow size distribution with d_32_ = 92 ± 13 nm (Figure 4), close to the nominal pore size of the membrane used for their extrusion (see Experimental part). The results for bare SLS, SLES, ALS, CAPB and soapwort solutions at 5% dry mass content (Figure 3A) point to the presence of large aggregates (possibly worm-like micelles) in solutions of the anionic surfactants (SLS, SLES, ALS), with d_32_ in the range of 370–630 nm, and much smaller (d_32_ = 6 nm)—for the amphoteric CAPB (Figure 4). On the other hand, the aggregates present in the soapwort extract (SAP) are much more polydisperse and span a range from 900 to 1500 nm (d_32_ = 1178 ± 165 nm). The latter consists mainly of triterpenoid saponins (total saponin content 73.90 ± 2.08% [38]) but may also contain several other water-soluble biomolecules present in the plant material, e.g., polyphenols, tannins, sugars, etc. This complex and heterogeneous mixture gives rise to a much broader particle size distribution than observed for the synthetic surfactants, where only the respective homologs differing slightly in the number of methylene or methoxy groups are present.

The model liposomes were completely solubilized in the given range of (bio)surfactant concentration only in the case of CAPB, where the peak at d_32_ = 92 nm disappeared completely, leaving only very small aggregates of d_32_ = 6 nm. Most likely, they correspond to the CAPB micelles with the lipids solubilized in their hydrophobic cores. For the other synthetic surfactants, the large particles (d_32_ > 350 nm) were replaced with those of sizes corresponding to the original liposomes, despite the total lipid concentration being as low as 1 × 10^−2^%. These peaks vanished only at dry mass content exceeding 5% (for example, between 5 and 10% in the case of SLS (not shown)). This observation is rather surprising because in molar terms (taking into account the differences in molecular weight between the lipids constituting the liposomes and the surfactant molecules) the number of lipid molecules is thus about two orders of magnitude smaller than that of the (bio)surfactants. Given the overwhelming excess of the surfactant molecules over the lipids, the resistance of the liposomes to the anionic synthetic surfactants is surprising, especially in view of other works reporting lipid solubilization by synthetic surfactants. It should be stressed, however, that most studies on liposome solubilization reported so far deal only with nonionic surfactants, especially Triton X-100 [39,40,41,42].

The high resistance of the DPPC/cholesterol liposomes to the anionic surfactants is also surprising in view of our previous results on solubilization of monolayers with the same lipid composition upon contact with SLS, ALS and SLES introduced into the subphase [21]. These monolayers were completely solubilized at the surfactant concentration as low as 1%, i.e., much lower than for the bilayers. Only for the amphoteric CAPB, the solubilization capacity is comparable towards the mono- and bilayers (both the monolayer and the liposomes vanished in presence of CAPB). In contrast, 1% SAP solution did not solubilize the same monolayer, although clear signs of incorporation in the lipid structure could be noted [21]. The same solution at 5% dry mass increased the size of the liposomes from below 100 nm to over 300 nm (note the disappearance of the original large structures, d_32_ = 1178 ± 165 nm, observed in bare SAP solutions). Such swelling could be easily explained by the analogous incorporation of the SAP components (especially saponins) into the liposomes, as observed previously in the case of monolayer penetration. Analogous mixed lipid-saponin aggregates have already been reported and well-characterized, e.g., for a purified saponin from horse chestnut seeds, escin [43,44].

Taking into account the overall effect on mono- and bilayers of DPPC and cholesterol mixed in a 7:3 mol/mol ratio, the present set of (bio)surfactants can be divided into three groups:

(1) Anionic (SLS, ALS, SLES), which solubilizes well with the monolayers but not the bilayers.
(2) Nonionic (CAPB), which solubilizes well with both mono- and bilayers.
(3) Biosurfactant (SAP), which penetrates without solubilization the monolayers at 1% dry mass, and probably penetrates bilayers, increasing their size (without solubilization).

The DLS study using the model DPPC/cholesterol liposomes proved that the aggregates present in all tested (bio)surfactants at 5% dry mass content disappear in presence of the lipids. On the other hand, a direct proof of the liposomes disappearance below 5% dry mass could be gathered only for CAPB; for the anionic synthetic surfactants (SLS, ALS, SLES) and for the soapwort extract (SAP), the DLS could not provide a clear proof of liposome solubilization up to the (bio)surfactant concentration of 5%. Thus, based on the DLS results, simple lipid solubilization could unequivocally explain the observed cytotoxicity towards the cell lines only in the case of CAPB. Verification of this mechanism for the remaining (bio)surfactants requires a different approach. To this aim, we compared their emulsification capacity test using two model oils—one mimicking the composition of sebum secreted by human skin (olive oil) and one representing a model greasy dirt difficult to remove (engine oil). The similarity of the composition of olive oil to human sebum results from the high content of squalene, β-sitosterol and fatty acids [45]. Analogous tests are typically employed to compare the washing abilities of different detergents, so in addition to the mechanistic questions, they will provide reliable grounds for the comparison of the practical usefulness of SAP and its synthetic counterparts. Surfactants whose washing properties are too strong may not only remove dirt from the skin surface but also its protective hydro-lipidic layer, which may lead to excessive water loss by the skin and its excessive drying [15,46]. In this context, an ideal surfactant would show a minimum emulsification capacity towards the olive oil and a maximum one—towards the engine oil.

**Figure 5:**
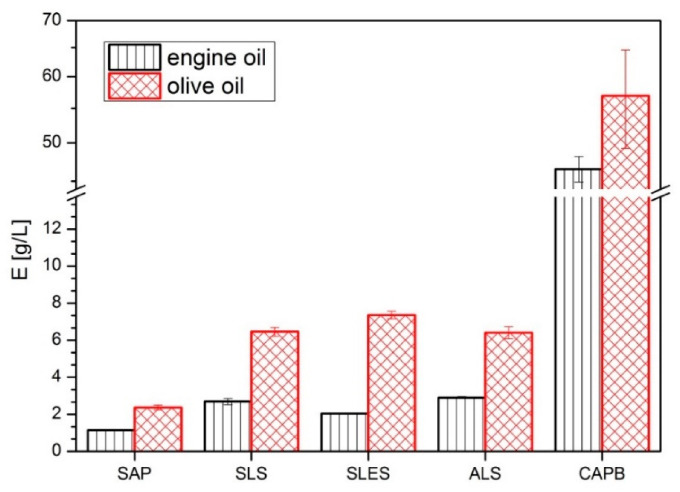
Oil emulsification capacity (E) of the soapwort extract (SAP) and synthetic surfactants (SLS, SLES, ALS, CAPB) towards the olive and engine oils, defined as a maximum weight of oil that can be emulsified in 1 L of 1% (bio)surfactant solution at room temperature.

The oil emulsification capacity results (E) for both oils collected in Figure 5 show that all (bio)surfactants are more efficient towards the olive oil (higher E) and that the order of their efficiency is the same for both oils. CAPB is clearly the most efficient oil emulsifier (E = 57 ± 8 g/L for olive oil and E = 46 ± 2 g/L for engine oil), the anionic showing intermediate capacities (E ≥ 6.5 g/L and E ≥ 2 g/L for the olive and engine oil, respectively) and SAP being the least efficient emulsifier (2.4 ± 0.13 g/L, 1.2 ± 0.01 g/L). The results agree with the observations from the liposome study, confirming the strongest oil solubilizing ability of CAPB. The anionic surfactants (SLS, ALS, SLES) are also efficient oil emulsifiers, so it is likely that their cytotoxic activity towards the cell lines is in fact related to their detergent activity (membrane lipid solubilization). On the other hand, this mechanism does not seem likely in the case of SAP, therefore in the next part of the study, we proceed to compare the protein solubilization potential of the (bio)surfactants. This will allow us to verify the second possible cytotoxicity pathway—via protein solubilization and at the same time compare their skin-irritating potential.

One of the main roles of keratinocytes in the skin is the secretion of keratins—a family of fibrous structural proteins. Keratin is released from the late keratinocytes (corneocytes), constituting the “bricks” of the outermost epidermis layer. Thus, in addition to lipid removal (discussed above), skin irritation can be triggered also by the solubilization of keratin, either from alive (keratinocytes) or dead (corneocytes) cells. The same applies to all other proteins crucial to the skins’ functioning, including also those present in the skin cells’ membranes. Therefore, in the next part of the study, we compared the solubilization potential of the tested (bio)surfactants towards two model proteins often employed for testing the skin irritation potential of cosmetics or household chemicals: bovine serum albumin [47,48] and corn zein [49,50,51].

**Figure 6:**
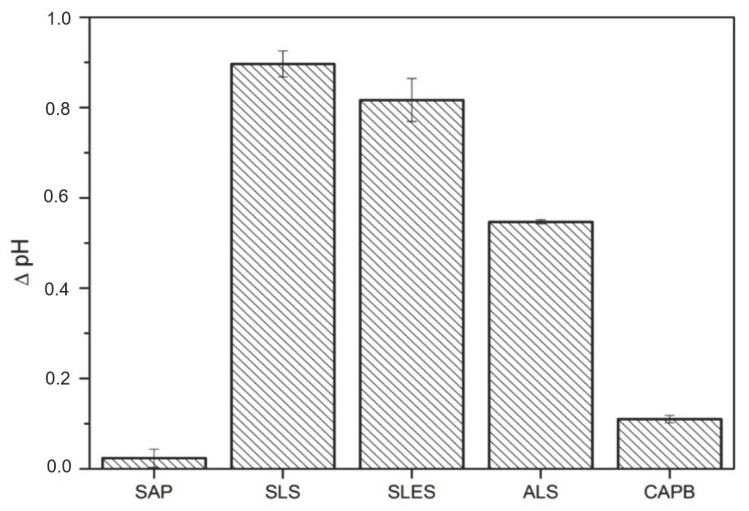
pH increases in BSA solution (∆ pH) incubated with the soapwort extract (SAP) or synthetic surfactants (SLS, SLES, ALS, CAPB), 5% concentration of the dry weight, after 2 h incubation with albumin at room temperature.

Upon surfactant binding, many proteins, including bovine serum albumin (BSA), undergo partial denaturation which is often accompanied by the consumption of protons from the environment. The consequent increase in the pH of the solution is a relative measure of the extent of surfactant binding and denaturation which is often linked with skin irritation [47]. The results presented in Figure 6 show that the anionic surfactants increase pH to the highest extent, in agreement with their well-known high protein-denaturation potential [48,52] and literature reports on their ability to increase pH of BSA solutions [53]. The order of the BSA-induced pH increase (ΔpH) agrees with the commonly accepted order of the anionics’ harshness: SLS (0.9) > SLES (0.8) > ALS (0.5). Depending on the alkyl chain length, for three sodium alkylsulfate homologs with C_8_, C_10_ and C_12_ alkyl chains, the pH increase between 0.3 and 1.5 has previously been reported [47]. On the other hand, CAPB and SAP are located on the other extreme of the surfactant-induced BSA denaturation. The negligible pH change in the case of CAPB is related to its zwitterionic character and has already been reported [47]. However, the lack of pH change for the soapwort extract is surprising given the anionic character of many saponins [54]. Overall, the results from Figure 6 do not support the hypothesis of the protein-solubilization pathway of the skin cells cytotoxicity in the case of SAP and CAPB, in contrast to SLS, SLES and ALS.

**Figure 7:**
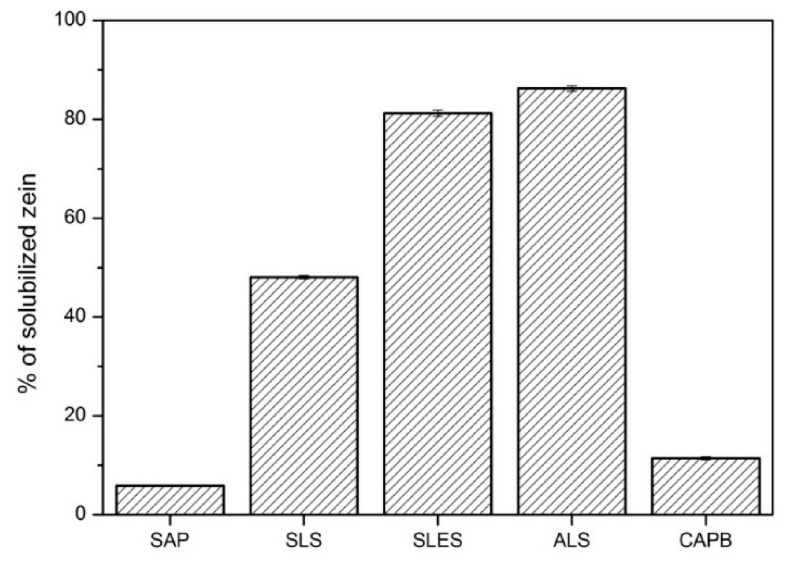
Percentage of solubilized zein in soapwort extract (SAP) or synthetic surfactants (SLS, SLES, ALS, CAPB), 5% concentration, after 1 h incubation with corn zein at 35 °C.

In contrast to BSA, corn zein in its native form is insoluble in water. It can be, however, solubilized as a consequence of surfactant-induced denaturation [51], especially for anionics [55]. A classical zein test consists of the determination of nitrogen present in solution (Kjeldahl method, elemental analysis) which is a basis of determination of so-called zein number (ZN, defined as the amount of zein, expressed in mg nitrogen, dissolved by a surfactant in 100 mL of a surfactant solution). In this contribution, we propose, however, a much simpler procedure, based on simple dry mass increase in a (bio)surfactant solution exposed to an excess of solid zein powder. Any increase in the dry mass must originate from zein solubilization by the given (bio)surfactant. The percentage of solubilized zein shown in Figure 7 shows similar general trend as in the case of BSA (Figure 6): anionic >> CAPB (11 ± 1%) > SAP (6 ± 1%), although the intragroup trend is the opposite for the anionics: ALS (86 ± 1%) > SLES (81 ± 1%) > SLS (48 ± 1%). For comparison, only 0.4% of zein can be solubilized in pure water under the same conditions. The method was validated by analyzing the nitrogen content (CHNS, Elementar Vario EL III) in the SLS-solubilized and water-solubilized samples, giving ZN = 564 and 9.4 for SLS and water, respectively. These values agree very well with the literature data (500 ± 20 and 20 ± 10, respectively) [51]. Additionally, the weak zein solubilization potential of CAPB observed in our results agrees with the literature reports (ZN < 40 mg/100 mL [56]. Thus, based on the results from Figure 7 we can conclude that the soapwort extract does not behave like anionic synthetic surfactants also with respect to zein, confirming its mild anionic character towards model proteins. This further strengthens the hypothesis that the cytotoxicity of SAP and CAPB towards the skin cells is not due to protein solubilization, in contrast to the anionic surfactants.

## 3. Materials and Methods

Dried roots of soapwort (*Saponaria officinalis* L.) were purchased from “Dary Podlasia,” herbal provider (Bielsk Podlaski, Poland). The aqueous extract was prepared by decoction: pouring cold water on the dry plant material, heating till boiling and maintaining in boiling for 15 min, followed by cooling down to room temperature. The extract was filtered using a Colombo 18 OIL filter press (Rover Pompe, Polverara, Italy) using paper plates with pore sizes of 15 µm, 11 µm, 6 µm and 3 µm. It was then dried using a YC-015A lab spray dryer (Pilotech, Shanghai, China). The chamber temperature was set to 120 °C and the outlet temperature (effective drying temperature) equaled typically 70 °C. The dried extract (SAP), with total saponin content of 110 ± 2.4 mg/g of dry mass (determined using UPLC-MS, as described in ref. [45]) was stored at room temperature and was dissolved in phosphate buffer (pH = 7, I = 10^−3^ M) immediately before the measurements.

Synthetic surfactants: sodium lauryl sulfate (SLS), sodium laureth sulfate (SLES), ammonium lauryl sulfate (ALS) and cocamidopropyl betaine (CAPB) were kindly provided by PCC Exol (Brzeg Dolny, Poland). Their solutions in phosphate buffer (pH = 7, I = 10^−3^ M) were prepared analogously to those of plant extract, in a way to produce the required final dry mass content.

1,2-Dipalmitoyl-sn-glycero-3-phosphocholine, DPPC (purity ≥ 99%, semisynthetic) and cholesterol, CHOL (purity ≥ 99%) were obtained from Sigma-Aldrich, Poznan, Poland. All lipids for monolayer deposition were dissolved in chloroform (purity ≥ 99.8%) and methanol (purity ≥ 99.9%), 9:1, both purchased from Sigma-Aldrich, Poznan, Poland, and used without any further purification. Milli-Q water (Merck Millipore, France) was used to prepare all solutions. The phosphate buffer, all synthetic surfactants solutions and reconstituted extract of soapwort were filtered through a 0.22 µm syringe filter prior to the measurements.

Liposomes mimicking the lipid composition of the cell membrane of keratinocytes were prepared by hydration [57] of DPPC/cholesterol (molar ratio 7:3) [58] films at 2 mg/mL. The lipids were dissolved in chloroform and methanol (volume ratio 9:1) and the solvent was evaporated with a stream of compressed air. Next, 1 mL of the phosphate buffer (pH = 7, I = 10^−3^ M) was added and the mixture was heated under warm tap water, shaken from time to time in a vortex (Lab dancer, IKA, Königswinter, Germany) to facilitate detachment of the lipid film from the walls of the vessel. The suspension was then sonicated using a Sonopuls HD 2070 setup (Bandelin, Berlin, Germany) for 15 min, extruded using a mini-extruder (Avanti Polar Lipids, labaster, AL, USA) through a 100 nm filter membrane (33 times) and diluted 20-fold with the respective surfactants or soapwort extract solutions in the phosphate buffer (pH = 7, I = 10^−3^ M). The final dry mass of the surfactant in the mixture with liposomes was set to 0.001%, 0.04%, 0.2%, 1%, 5% (*w*/*w*). The size distribution of the liposomes after exposition to the (bio)surfactants was evaluated by dynamic light scattering (DLS) [59] using a Zetasizer HS3000 (Malvern, UK) for 2 h, the time sufficient to reach stable readings. The results are presented as size distribution diagrams and the Sauter mean diameter (d_32_) values [60], which describes the diameter of a particle with the same volume to surface ratio, was calculated using the following equation:

$$d_{32}=\frac{\sum N_{i}\times d_{i}^{3}}{\sum N_{i}\times d_{i}^{2}}$$

where *N_i_*—the signal intensity of the *ith* particle diameter [%]; *d_i_*—the diameter of the *ith* particle [nm].

The DLS measurements uncertainty (14%) was established by determining the average value of the Sauter mean diameter (d_32_) of 4 randomly chosen samples, each of which was measured at least six times.

The HaCaT (Human keratinocyte, ThermoFisher, Waltham, MA, USA), and A375 (Human skin malignant melanoma cells, ATCC) cell lines were chosen as models of the human skin cells. The HaCaT and A375 cell lines were cultured in a complete DMEM High Glucose medium (Dulbecco’s Modified Eagle Medium, Biowest, France) supplemented with 10% (*v*/*v*) fetal bovine serum (FBS, ThermoFischer, Waltham, MA USA), 1% of penicillin and streptomycin (Sigma-Aldrich, Poznan, Poland) (*v*/*v*), and 1% of l-glutamine (Sigma-Aldrich, Poznan, Poland) (*v*/*v*). The cells were maintained under 5% CO_2_ at 37 °C and 95% humidity.

The biological activity of the tested surfactants and the plant extract against the HaCaT and A375 cell lines was evaluated by measuring the cells’ viability after their exposure to the (bio)surfactant solutions in the concentration range from 0.01 to 50 mg/mL. The culture medium alone was used as a control. Cell viability was evaluated using a colorimetric assay for assessing the cell metabolic activity (MTT test). The test is based upon the NAD(P)H-dependent cellular oxidoreductase enzymes activity which, under defined conditions, may reflect the percentage of cellular viability. The enzymes are capable of reducing a tetrazolium dye 3-(4,5-dimethylthiazol-2-yl)-2,5-diphenyltetrazolium bromide (MTT) to purple-colored water-insoluble formazan. The cells were seeded in the standard 96-well plates at a density of 1·10^5^ cells per ml and incubated to assure their adhesion to the culturing surface. Then, the medium was removed and replaced with a series of solutions of the tested extract or synthetic surfactants (0.01–50 mg/mL). The solutions were added to the multi-well plate in eight replicates and the cells were incubated under 5% CO_2_ at 37 °C for 24 h. The controls were carried out in the absence of tested substances (the cell culture was incubated only with the respective fresh medium). In order to verify the influence of the tested (bio)surfactants on the cell culture, the cells were treated with MTT (Sigma-Aldrich, Poznan, Poland) solution (0.5 mg/mL in phosphate-buffered saline (PBS) (Sigma-Aldrich, Poznan, Poland; 100 µL per well). The cells were protected from light and incubated with the MTT solution for the next 3 h. Then, the supernatant was carefully removed and the formed violet formazan crystals were dissolved in dimethyl sulfoxide (DMSO, Sigma-Aldrich, Poland; 100 µL per well). The absorbance of the formazan solutions was measured at λ = 570 nm using a Multiwell Plate Reader Biotek Cytation 3 (Biotek, Winooski, VT, USA). The results were expressed as a percentage of viability in comparison to the control groups, according to the formula below.

Cells’ viability = *A_i_/A_c_* × 100%,

where *A_i_*—average absorbance of tested group; *A_c_*—average absorbance of control group.

On the basis of viability assays results, the values of EC_50_ parameter were calculated, using an online tool AAT Bioquest (https://www.aatbio.com/tools/ec50-calculator) (accessed on 1 August 2021).

The albumin denaturation test was performed using a 2% *w*/*w* aqueous solution of bovine serum albumin (Sigma-Aldrich, Poznan, Poland) and 10% *w*/*w* aqueous solutions of the tested (bio)surfactants [47]. The pH of both solutions was fixed with sodium hydroxide and citric acid solutions at pH = 5.5. Next, the albumin and the (bio)surfactant solutions were mixed in equal proportions (5 mL of each) and stirred (200 rpm/min) at room temperature for 2 h, followed by the pH measurement. The results are presented as a mean difference between the initial pH (5.5) and that determined after 2 h for 3 independent samples.

The zein solubility test was carried out according to the method described by Götte [61] with some modifications. A total of 10 mL of an aqueous solution of the tested (bio)surfactant (5% *w*/*w*) was added to 0.5 g of corn zein (Sigma-Aldrich, Poznan, Poland). The mixture was stirred for 60 min at 35 °C, followed by centrifugation at 5000 rpm/min for 20 min and filtering through a soft filter paper (Chemland, Stargard, Poland). The dry matter in the supernatant was determined using a moisture analyzer (Axis ATS 120, Poznan, Poland). The results are presented as an average value of zein loss into the solution for three independent samples.

The oil emulsification capacity test [62] was performed using two model oils: food-grade olive oil (purchased in a local grocery store in Warsaw, Poland) and engine oil (Elf evolution, Lesquin, France). In a 10 mL flask, one drop of the oil (approx. 10 mg) stained with Sudan IV Red (Sigma Aldrich, Poznan, Poland; 85 mg/100 g oil) was precisely weighed. Then, the tested (bio)surfactant solution (1%, *w*/*w*) was added, and the mixture was vigorously stirred for about 30 s (IKA, lab dancer). The aqueous solution was added initially in portions of 1 mL and when the mixture became turbid—of 200 µL until the oil was dispersed. Each experiment was conducted in at least three repetitions. The results are presented as the maximum weight of the oil that can be emulsified in 1 L of the (bio)surfactant solution (1%, *w*/*w*).

## 4. Conclusions

The present study compares cytotoxicity towards normal keratinocytes and human melanoma cell line of the soapwort (*Saponaria officinalis* L., SAP) extract and four synthetic surfactants (three anionics: sodium lauryl sulfate (SLS), sodium laureth sulfate (SLES), ammonium lauryl sulfate (ALS) and one amphoteric: cocamidopropyl betaine (CAPB)). SAP did not show a cytotoxic effect on normal human keratinocytes (HaCaT) in the dry mass range up to 0.039%, in contrast to all four tested synthetic surfactants which were toxic already above 0.02%. At dry mass contents below 0.6%, the soapwort extract is less harmful than SLS, SLES, CAPB, or even the gentlest from this group—ALS. Comparison of the results suggests that the cancerous cells (A375) are in general more sensitive to the effect of the synthetic surfactant. In that respect, the synthetic surfactants could perhaps be considered as potentially promising for anticancer purposes, if not the fact that at the same time they are also toxic to the normal cells. The opposite trend could be observed for the soapwort extract, where the toxicity appeared at a much lower dry mass content for HaCaT (0.002%) than for A375 (0.078%). The soapwort extract is thus not a good candidate for the treatment of human melanoma (but still remains a very promising gentle skin cleansing agent, though). This observation might be somehow surprising given the numerous literature reports on the potential anticancer activity of many saponins [22,63,64]. On the other hand, the literature reports comparing the effect of the same saponin (or saponin mixture) on normal and cancerous cells are still rather scarce [65]. In other words, there is not much scientifically-sound proof confirming that saponins show general selectivity towards cancerous cells. Our recent results suggest that their selectivity might in fact be dictated mostly by the cells’ cholesterol content. We have shown that the mixture of saponins from a Chilean tree *Quillaja saponaria* Molina (known as Quillaja bark saponis, QBS) is cytotoxic to the cancerous lung cells (A549), but even higher toxicity was observed towards the normal lung cells (MRC-5), which were more abundant in total cholesterol [66].

In order to gain a deeper understanding of the origin of the observed differences in cytotoxicity towards human skin cells, we compared the liposome solubilization (DPPC/cholesterol) and lipid emulsifying capacity (olive oil, engine oil) as well as protein solubilization (albumin and zein tests) of all (bio)surfactants. This allowed us to conclude that the increased cytotoxicity of SAP observed at higher dry mass contents (>0.6%) is most likely not a consequence of simple lipid and/or protein solubilization, as in the case of the synthetic surfactants. The surface-active components of SAP, mostly triterpenoid saponins, show much smaller lipid solubilizing potential than any of the tested synthetic surfactants. While this limits the SAP’s efficacy in dirt removal in comparison to the synthetic counterparts, one may speculate that cosmetic formulations based on SAP should be gentler to the skin lipids (both constituting the keratinocytes membranes and the *stratum corneum*’s lipid matrix), especially when compared to CAPB. Even more drastic differences could be noted in the protein-solubilizing potential, where SAP was found by far the least harmful. The present cytotoxicity assay confirmed that replacing the currently employed synthetic surfactants with the soapwort extract might be beneficial for cosmetics, pharmaceutical and household formulations when mildness and naturalness are especially important.

## Figures and Tables

**Table 1:** EC_50_ values expressed as dry mass content [%] for (bio)surfactants from Figure 2.

Cell Line	EC_50_ [Dry Mass Content, %]				
	SAP	ALS	SLES	SLS	CAPB
HaCaT	0.3999	0.0210	0.0004	0.0012	0.0007
A375	0.1348	0.0259	0.0016	0.0013	0.0011

## Data Availability

The data presented in this study are available on request from the corresponding author.

## Sample Availability

Samples of the (bio)surfactants are available from the authors.
